# A retrospective cohort study of the clinical safety of endobronchial ultrasound in patients with superior vena cava obstruction

**DOI:** 10.1097/MD.0000000000042969

**Published:** 2025-06-13

**Authors:** Justin Siew Yoong Tu, Ashleigh Witt, Christina Giudice, Daniel Steinfort, Stefan Heinze, Kanishka Rangamuwa

**Affiliations:** a Department of Respiratory Medicine, Royal Melbourne Hospital, Melbourne, Australia; b Department of Medicine, University of Melbourne, Melbourne, Australia; c Department of Medical Imaging, Royal Melbourne Hospital, Melbourne, Australia; d Department of Respiratory Medicine, The Northern Hospital, Melbourne, Australia.

**Keywords:** endobronchial ultrasound-guided transbronchial needle aspiration (EBUS-TBNA), interventional pulmonology, superior vena cava obstruction (SVCO), superior vena cava syndrome (SVCS)

## Abstract

Superior vena cava obstruction (SVCO) is a radiographic finding in patients with mediastinal masses, which may be accompanied by features of superior vena cava syndrome (SVCS). SVCS is associated with physiologic impairments that increase procedural risk. Data regarding the risk of complications following endobronchial ultrasound-guided transbronchial needle aspiration (EBUS-TBNA) in patients with SVCO is limited. To assess whether rates of procedural complications from EBUS-TBNA are increased in patients with SVCO. A retrospective observational study was performed on all patients undergoing EBUS-TBNA by the respiratory department at major Australian tertiary center from January 1 to December 31, 2021. Imaging review identified radiographic SVCO, which was classified by anatomical location and degree of obstruction following a grading system previously described in the literature. Clinical features of SVCS were noted, alongside and procedural outcomes and complications. The primary outcome for this study was the rate of complications. Data was assessed with simple statistics and chi-square analysis. 245 linear EBUS procedures were performed. Radiographic SVCO of moderate severity or greater (>50% obstruction) was present in 11 (4%) patients, mild severity (<50% obstruction) in 13 (5%), indeterminate but likely in 4 (2%), indeterminate but unlikely in 20 (8%), and absent in 197 patients (80%). Rates of major complications were low across the cohort (7% in patients with SVCO, 8% in patients with SVCO), and were independent of SVCO (*P* = .34). Amongst the 24 patients with SVCO, the original radiology reports mentioned obstruction in only 4 patients, and overall clinical features of SVCS was rarely documented or assessed. SVCO is underreported and under-assessed in our cohort of patients both clinically and radiologically, however SVCO does not appear to be associated with a greater risk of complications following EBUS-TBNA. This study demonstrates that EBUS-TBNA can safety be performed in patients with radiological SVCO.

## 1. Introduction

Superior vena cava syndrome (SVCS) describes a group of signs secondary to obstruction of blood flow through the superior vena cava (SVC).^[1]^ It is characterized clinically by edema in the upper extremities with neurological and laryngopharyngeal symptoms, the severity of which depends on the location and the speed of onset.^[1]^ In contrast, superior vena cava obstruction (SVCO) is an anatomical definition that can be demonstrated with narrowing of the SVC diameter on contrast-enhanced computed tomography (CT).^[1–4]^ In cases where the progression of SVCO is subacute, this may not manifest clinically as SVCS due to the presence of collateral venous systems.^[1]^

SVCS is caused by malignancy in 70% of cases, with other causes including iatrogenic obstruction, radiation fibrosis and infection.^[1,5]^ Tissue diagnosis via biopsy is critical to determine causative pathology and is required to guide targeted treatment in this life-threatening clinical scenario. Historically, bronchoscopic procedures have been considered to have a high risk of procedural complications in patients with SVCS.^[6]^

Endobronchial ultrasound-guided transbronchial needle aspiration (EBUS-TBNA) is the preferred method of obtaining a tissue diagnosis in mediastinal and hilar masses and lymph nodes.^[7,8]^ Whilst it has been demonstrated to have both a high diagnostic yield and safety profile in general, there exists very limited data on the safety profile of EBUS-TBNA in SVCS. A case series by Wong et al of EBUS-TBNA in SVCS demonstrated a transient increase in oxygen requirement in 28% of cases with no increased bleeding or mortality risk.^[5]^ A comparative study by Boily-Daoust et al also reported low rates of complications when comparing the safety of rigid bronchoscopy, EBUS-TBNA and transthoracic needle biopsy.^[9]^

A scoring system for severity of SVCS has been proposed by Yu et al^[10]^ though limited studies suggest no correlation between SVCS severity and the incidence of procedural complications.^[8]^

We performed a retrospective cohort study to assess the incidence of SVCO amongst patients undergoing EBUS-TBNA and its relation to clinical SVCS and complication rates with EBUS-TBNA.

## 2. Materials and methods

This was a retrospective cohort study of all patients undergoing EBUS-TBNA between January 1, 2021 and December 31, 2021 at a major a tertiary lung cancer referral center in Melbourne, Australia. We reviewed medical records to assess for baseline characteristics, diagnosis of or symptoms suggestive of SVCS pre-procedure, tissue diagnosis, type of procedure, and complications.

EBUS-TBNA is performed under conscious sedation administered by a consultant anesthetist. EBUS procedures are performed by 5 consultant respiratory physicians and a pulmonology fellow acting under direct supervision.

**Figure 1.**
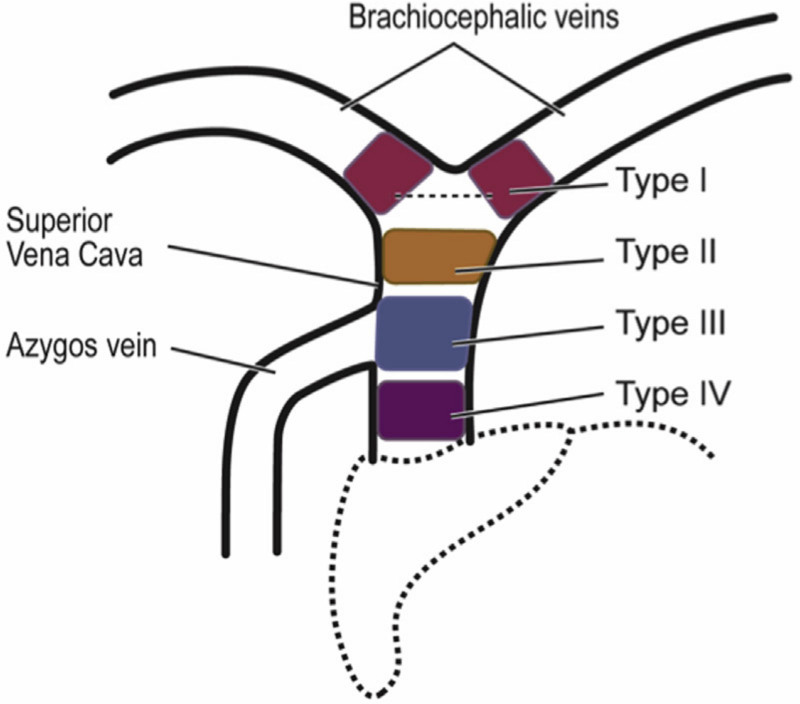
Anatomical classification of superior vena cava obstruction. Diagram obtained with permission from Azizi et al 2020^[1]^.

Pre-procedural CT imaging was then reviewed by an independent radiologist for features of SVCO. The severity of the obstruction was graded according to the system proposed by Azizi et al,^[1]^ summarized in Figure 1 and Table 1. This system assigns the anatomical location of the lesion as Type I to Type IV, and the degree of obstruction as grade A (moderately occluded) through grade C (totally occluded). A further category of “mild obstruction” was introduced as a category by the authors for patients who had evidence of SVC narrowing <50%. CT images without any features of obstruction were classified as “no obstruction.”

**Table 1:** Classification of superior vena cava obstruction.

Lesion location	Severity			
	Mild obstruction (<50%)	Grade A: moderate-severe (50–90%)	Grade B: pre-occlusive (>90%)	Grade C: totally occluded (100%)
Type I: Bilateral brachiocephalic vein occlusion with or without supra-azygous SVC	I mild	Ia	Ib	Ic
Type II: Supra-azygous SVC without brachiocephalic involvement	II mild	IIa	IIb	IIc
Type III: Azygous SVC	III mild	IIIa	IIIb	IIIc
Type IV: Infra-azygous SVC	IV mild	IVa	IVb	IVc

Table adapted from Azizi et al 2020.^[1]^

SVC = superior vena cava.

**Figure 2.**
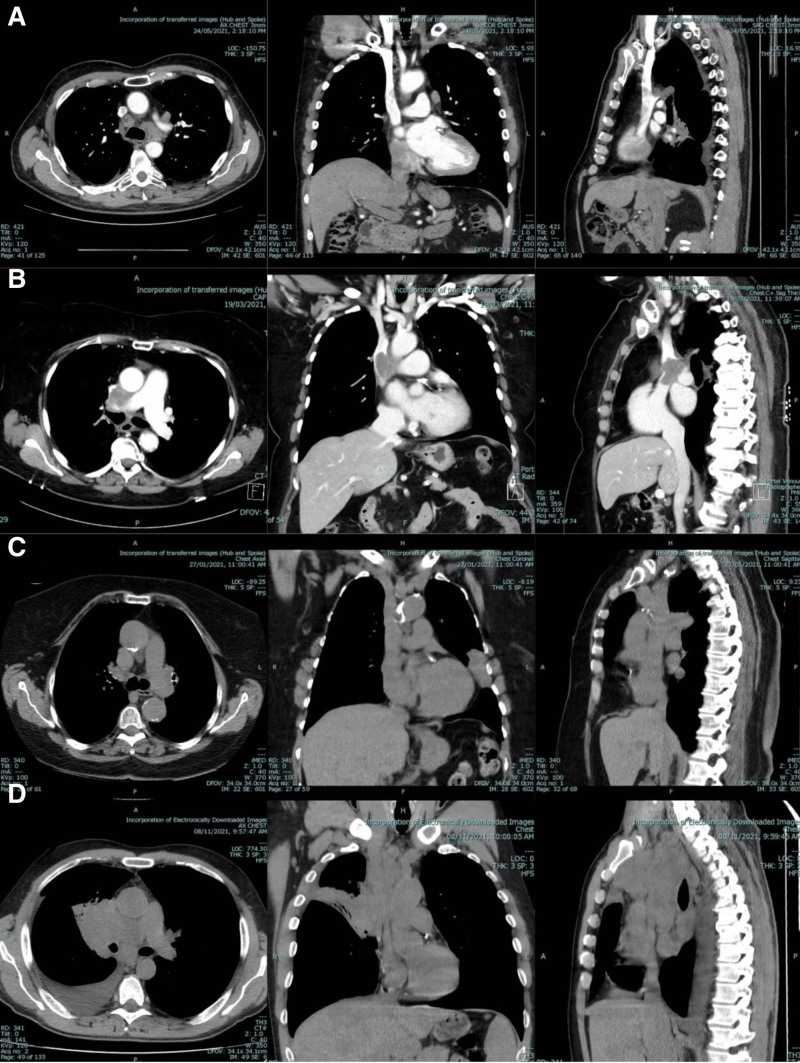
Examples of the presence and absence of SVCO in CT imaging with and without contrast. (A) Example of contrast-enhanced CT chest without obstruction; (B) example of contrast-enhanced CT chest with obstruction; (C) example of non-contrast CT chest without obstruction; (D) example of non-contrast CT chest with likely obstruction. CT = computed tomography, SVCO = superior vena cava obstruction.

Whilst all patients at our center routinely undergo CT scans of their chest and positron emission tomography before their EBUS procedure, CT is not always performed with IV contrast. In patients whose pre-procedure imaging was a non-contrast CT or positron emission tomography-CT, assessment and grading of SVC obstruction were at times limited. In these cases, available imaging was assessed for features suggestive of SVC obstruction, such as a mass encasing or adjacent to the SVC, involving the immediate pericaval mediastinal fat, and/or distortion or displacement of the SVC contour, with examples provided in Figure 2. Based on these imaging findings, these scans were then classified as “possible obstruction,” and “unlikely obstruction.”

Complications were documented by reviewing procedural and inpatient documentation at our center. For patients who were referred from a peripheral center, external medical records were reviewed and, if necessary, discussions with referring physicians were held to document delayed complications and 30-day readmission rates accurately. Complications were considered major if they resulted in escalation of care. Moderate bleed was defined as bleeding requiring intubation of the segment with bronchoscope or administration of cold saline or adrenaline. All statistical analyses were performed using R (version 4.0.2, R Foundation for Statistical Computing, Vienna, Austria). Pearson’s chi-square test of independence was performed to assess rates of complications in patients with and without SVCO.

This project was approved by the Royal Melbourne Hospital Office for Research Ethics & Governance team (QA2022101)

## 3. Results

Between January 1, 2021 and December 31, 2021, 245 EBUS-TBNA procedures were performed with 11 patients also undergoing confirmatory EUS-B-FNA. The mean age of patients was 64.8 years (range 32–86), with 69% of patients (n = 142) male. During the periprocedural period, 12% of patients (n = 30) were continued on aspirin, 1% (n = 2) on clopidogrel, and none on an anticoagulant, which is further detailed in Table S1, Supplemental Digital Content, https://links.lww.com/MD/P231.

From the 245 patients, radiographic SVCO of moderate severity or greater (>50%) was present in 4% (n = 11) of patients, of mild severity in 5% (n = 13), indeterminate but likely in 2% (n = 4), indeterminate but unlikely in 8% (n = 20), and absent in 80% (n = 197). Demographic data of patients with radiological evidence of SVCO are summarized in Table 2.

**Table 2:** Patients with radiological evidence of obstruction (n = 24).

	>50% obstruction	<50% obstruction (mild)
Patient data		
n	11	13
Age-mean (range)	61.8 (35–81)	58.7 (32–74)
Gender (male)	27% (n = 3)	62% (n = 8)
Weight (kg)	70.0	67.8
Height (m)	1.6	1.7
BMI (kg/m^2^)	25.1 (18.6–31.1)	24.1 (16.2–32.3)
Respiratory comorbidities		
Asthma	18% (n = 2)	8% (n = 1)
COPD	27% (n = 3)	31% (n = 4)
Interstitial lung disease	0% (n = 0)	0% (n = 0)
Diagnosis		
Primary lung malignancy		
Small cell lung cancer	5	3
Lung adenocarcinoma	4	4
Squamous cell carcinoma	0	1
NSCLC NOS	0	1
Large cell neuroendocrine	0	1
Metastatic malignancy		
Other adenocarcinoma	1	0
Haematological malignancy		
B cell lymphoma	0	1
Follicular lymphoma	1	0
Non-tuberculosis granulomatous disease		
Sarcoidosis	0	2

BMI = body mass index, COPD = chronic obstructive pulmonary disease, NSCLC NOS = non-small cell lung cancer not otherwise specified.

Amongst the 24 patients found to have SVCO on imaging review, we found their original radiology report often did not mention SVCO or related phrases. Just 4 reports mentioned SVCO, with an additional 2 referencing features of SVCO without stating this. Within our cohort, no patients demonstrated complete radiographic occlusion of the SVC (i.e., grade C severity). Table S2, Supplemental Digital Content, https://links.lww.com/MD/P232 summarizes the spectrum of severity and anatomical obstruction observed in our patient cohort alongside rates of SVCO reporting.

No patients were diagnosed with SVCS, although we note that clinical assessment for this was rarely documented. One patient’s clinical record noted “SVC compression on CT, reports difficulty lying flat.” A further 2 patients are documented to have negative findings for an SVCS examination.

The rate of complications was low across the cohort (Table 3). Only 1 complication, that of a moderate bleed requiring local adrenaline for haemostasis, occurred in a patient with pre-occlusive grade B (>90%) SVC obstruction, affecting several branches of the SVC, with multiple collaterals. This was identified on the imaging report, although no clinical assessment of SVCS was documented. No relationship between SVCO and occurrence of complications was identified (*P* = .34).

**Table 3:** Complications associated with EBUS-TBNA , grouped by evidence of radiographic obstruction.

	Moderate to severe obstruction (>50%)	Mild obstruction (<50%)	Non-contrast imaging, with possible obstruction	Non-contrast imaging with unlikely obstruction	No radiographic obstruction	Overall
Cardiac arrhythmia	0	0	0	1	5 (2*)	6 (2*)
Persistent hypoxia	0	0	1	0	6 (2*)	7 (2*)
Other unplanned admission	0	0	0	0	1†	1
Moderate bleed	1	0	0	2	3	6
Readmission within 30 d	0	0	0	0	0	0
No complications	10	13	3	17	182	225

EBUS-TBNA = endobronchial ultrasound-guided transbronchial needle aspiration.

* Patients who were able to be discharged on the day of the procedure, following the management and resolution of their complications.

† Patient was discharged but represented to ED with a post-bronchoscopy fever.

## 4. Discussion

In our cohort, SVCO identified on imaging was not associated with an increase in bleeding, periprocedural complications or 30-day readmission rate. These findings align with previous findings,^[5,8]^ suggesting that EBUS-TBNA is a safe and viable procedure for patients with SVCO demonstrated on imaging. Consistent with previous studies, the vast majority of patients in this cohort ultimately received a diagnosis of primary lung malignancy.

Our review highlights that patients undergoing endobronchial ultrasound at our health service are not assessed for clinical signs of SVCS in any formal or routine way. Even where there was radiological indication of SVCO and this was documented in the radiologist’s report, only 50% of patients had a documented examination looking for signs of SVCS.

Interestingly, no patients in our cohort had SVCO with grade C severity (100% obstruction), it is unknown whether patients present symptomatically prior to this stage, or if patients with high-grade SVCO are instead directed towards alternative diagnostic pathway, or empiric management due to perceived anesthetic risk when discussed in the lung cancer multidisciplinary meeting. Further research into outcomes and diagnostic pathways in patients with high severity obstruction is needed, as there is a paucity of data on the safety of CT guided biopsy in SVCO.^[11,12]^

Our findings revealed variability in radiology reporting practices, with the majority of formal radiology reports not containing comment on SVCO, even when present on subsequent radiology review. This inconsistency likely reflects the absence of a standardized classification system, as well as interobserver variability and report-writing preferences. In cases where non-contrast CT was utilized as the pre-procedural imaging, the evaluation of SVCO was limited. This paper details features of possible SVCO on non-contrast imaging, namely the presence of a mass encasing or adjacent to the SVC, involving the immediate pericaval mediastinal fat, and/or distortion or displacement of the SVC contour. Given there is no recommendation for the pre-procedural imaging for EBUS-TBNA to be a CT with IV contrast, awareness of these features will help ensure cases of SVCO are detected. As these findings have not been formally validated, the current confirmatory diagnostic test would be a contrast-enhanced scan. Considering the findings of this study indicate that radiologically detected SVCO does not impact complication rate, performing such scanning should not delay the decision to proceed to EBUS-TBNA.

The primary limitations of this study are its retrospective design and reliance on previously documented examination findings. As noted, in cases where only non-contrast pre-procedural CT was available, the definitive assessment of SVCO was limited. Furthermore, as a single center study with a limited sample size, future studies assessing the generalisability of these findings will be valuable.

## 5. Conclusion

SVCO is not uncommon in a cohort of patients referred for EBUS-TBNA, but is underreported in radiology reports. Additionally, clinical assessment of SVCS was infrequent in our cohort of patients.

Despite this, our data demonstrates that EBUS-TBNA is a safe procedure in patients with SVCO. Further research is warranted to improve recognition of this condition in both radiological and clinical assessments.

## Author contributions

**Conceptualization:** Justin Siew Yoong Tu, Ashleigh Witt, Kanishka Rangamuwa.

**Data curation:** Justin Siew Yoong Tu, Ashleigh Witt.

**Formal analysis:** Justin Siew Yoong Tu, Christina Giudice, Stefan Heinze, Kanishka Rangamuwa.

**Investigation:** Justin Siew Yoong Tu, Ashleigh Witt, Christina Giudice, Stefan Heinze, Kanishka Rangamuwa.

**Supervision:** Ashleigh Witt, Daniel Steinfort, Kanishka Rangamuwa.

**Writing – original draft:** Justin Siew Yoong Tu.

**Writing – review & editing:** Justin Siew Yoong Tu, Ashleigh Witt.

## Supplementary Material


