# Supplementary material for: A retrospective cohort study of the clinical safety of endobronchial ultrasound in patients with superior vena cava obstruction
Source: Medicine (Baltimore). 2025 Jun 13;104(24):e42969. doi: 10.1097/MD.0000000000042969 (PMC12173306; doi:10.1097/MD.0000000000042969)
Supplement: Supplementary file 1 [file medi-104-e42969-s001.docx]

**Supplementary table 1: Patient demographics**

| **Supplementary table 1: Patient demographics** | | |
| --- | --- | --- |
| **Patient data** | |  |
|  | Age - mean (range) | 62.4 (14 - 87) |
|  | Gender (male) | 69% (n=142) |
|  | BMI (kg/m2) | 27.2 (16.2 - 53.3) |
| **Respiratory comorbidities** | |  |
|  | Asthma | 8% (n=19) |
|  | COPD | 24% (n=58) |
|  | Interstitial lung disease | 2% (n=4) |
| **Antiplatelet and anti-coagulant medication at the time of procedure** | |  |
|  | Aspirin | 14% (n=34) were on aspirin.  Of this, 88% (n=30) continued the medication during the procedure. |
|  | Clopidogrel | 4% (n=11) were on clopidogrel.  Of this, 22% (n=2) continued on the medication during the procedure. |
|  | Anticoagulant* | 9% (n=23) were on anticoagulant.  Of this, no patients continued the medication during the procedure |
|  | No antiplatelet/anticoagulation | 74% (n=182) |
| *Anticoagulant refers to warfarin, therapeutic clexane or direct acting oral anticoagulants | | |
