# Supplementary material for: A retrospective cohort study of the clinical safety of endobronchial ultrasound in patients with superior vena cava obstruction
Source: Medicine (Baltimore). 2025 Jun 13;104(24):e42969. doi: 10.1097/MD.0000000000042969 (PMC12173306; doi:10.1097/MD.0000000000042969)
Supplement: Supplementary file 2 [file medi-104-e42969-s002.docx]

**Supplementary table 2:** **Radiological reporting amongst patients with SVCO**

| **Radiological reporting amongst patients with SVCO** | | | |
| --- | --- | --- | --- |
| **ID** | **Segments involved** | **Maximal grade of severity** | **SVCO mentioned on initial report** |
| 90 | Brachiocephalic, Supra-azygous (mostly) and touch infraazygous | Type I (mild <50%) | No |
| 57 | Supra-azygous | Type II (mild <50%) | No |
| 68 | Supra-azygous | Type II (mild <50%) | No |
| 93 | Supra-azygous, azygous and bit of infra-azygous | Type II (mild <50%) | Possible obstruction |
| 32 | Supra-azygous | Type II (mild <50%) | No |
| 18 | Supra-azygous and azygous | Type III (mild <50%) | No |
| 63 | Azygous/Infra-azygous junction | Type III (mild <50%) | No |
| 94 | Azygous | Type III (mild <50%) | No |
| 105 | Mild compression of ayzgous SVC from bulky nodes | Type III (mild <50%) | No |
| 1 | Infra-azygous | Type IV (mild <50%) | No |
| 10 | Minor compression of supra/azygous. Mainly infra-azygous | Type IV (mild <50%) | No |
| 49 | Infra-azygous | Type IV (minimal) | No |
| 54 | Infra-azygous | Type IV (mild <50%) | No |
| 91 | Brachiocephalic, Supra-azygous (mostly) and azygous | Type Ia (~80%) | Yes |
| 114 | Bilateral brachiocephalics, supra-azygous, azygous and infra | Type Ib and Type IIIb | Yes |
| 110 | Supra-azygous | Type IIa | No |
| 95 | Supra-azygous, azygous and bit of infra-azygous | Type IIb | Yes |
| 80 | Supra-azygous, azygous and bit of infra-azygous | Type IIIa | No |
| 44 | Supra-azygous, azygous and bit of infra-azygous | Type IIIb | No |
| 34 | Azygous and Infra-azygous | Type IVa | No |
| 58 | Infra-azygous | Type IVa (80%) | No |
| 75 | Azygous and Infra-azygous | Type IVa (70%) | Yes |
| 76 | Mostly infra-azygous (little bit of azygous) | Type IVa | No |
| 35 | Infra-azygous | Type IVb | Possible obstruction |

**Figure Legends**

Fig. 1. Legend text.

Fig. 2. Legend text.

Figures should be mentioned in the manuscript text as follows:
Without round brackets:

“…shown in Figure 1…” or “…shown in Figures 1 and 4…” or “…shown in Figures 2–6…” always with capital letters and written out.

With round brackets:

“(shown in Fig. 1)” or “(shown in Fig. 1, 4)” or “(shown in Fig. 2–6)”, always abbreviated as “Fig.” followed by the number or numbers after a full stop and a space.

In the Legend:

“Fig. 1.” or “Fig. 1. a”, always abbreviated as “Fig.” followed by the number after a full stop and a space

Please note that the actual figures and all tables should be uploaded as separate items in their original file format.
